# Skin Irritation Testing beyond Tissue Viability: Fucoxanthin Effects on Inflammation, Homeostasis, and Metabolism

**DOI:** 10.3390/pharmaceutics12020136

**Published:** 2020-02-05

**Authors:** Renata Spagolla Napoleão Tavares, Silvya Stuchi Maria-Engler, Pio Colepicolo, Hosana Maria Debonsi, Monika Schäfer-Korting, Uwe Marx, Lorena Rigo Gaspar, Christian Zoschke

**Affiliations:** 1 School of Pharmaceutical Sciences of Ribeirão Preto, University of São Paulo, Av. do Café s/n, Monte Alegre, Ribeirão Preto, SP 14040-903, Brazil; renatasnt@usp.br (R.S.N.T.); hosana@fcfrp.usp.br (H.M.D.); lorena@fcfrp.usp.br (L.R.G.); 2 Clinical and Toxicological Analyses Department, School of Pharmaceutical Sciences, University of São Paulo, Av. Prof. Lineu Prestes, 748, Cidade Universitária, São Paulo, SP 05508-000, Brazil; silvya@usp.br; 3 Institute of Chemistry, University of São Paulo, Av. Prof. Lineu Prestes, 748, Cidade Universitária, São Paulo, SP 05508-000, Brazil; piocolep@iq.usp.br; 4 Institute of Pharmacy (Pharmacology & Toxicology), Freie Universität Berlin, Königin Luise Str 2+4, 14195 Berlin, Germany; monika.schaefer-korting@fu-berlin.de; 5 TissUse GmbH, Oudenarder Str. 16, 13347 Berlin, Germany; uwe.marx@tissuse.com

**Keywords:** antioxidants, epidermal growth factor receptor, interleukins, irritation, metabolism response, N-acetyltransferase, small heat and shock protein beta 1

## Abstract

UV light catalyzes the ozone formation from air pollutants, like nitrogen oxides. Since ozone reacts with cutaneous sebum lipids to peroxides and, thus, promotes inflammation, tumorigenesis, and aging, even broad-spectrum sunscreens cannot properly protect skin. Meanwhile, xanthophylls, like fucoxanthin, proved their antioxidant and cytoprotective functions, but the safety of their topical application in human cell-based models remains unknown. Aiming for a more detailed insight into the cutaneous fucoxanthin toxicity, we assessed the tissue viability according to OECD test guideline no. 439 as well as changes in inflammation (IL-1α, IL-6, IL-8), homeostasis (EGFR, HSPB1) and metabolism (NAT1). First, we proved the suitability of our 24-well-based reconstructed human skin for irritation testing. Next, we dissolved 0.5% fucoxanthin either in alkyl benzoate or in ethanol and applied both solutions onto the tissue surface. None of the solutions decreased RHS viability below 50%. In contrast, fucoxanthin ameliorated the detrimental effects of ethanol and reduced the gene expression of pro-inflammatory interleukins 6 and 8, while increasing NAT1 gene expression. In conclusion, we developed an organ-on-a-chip compatible RHS, being suitable for skin irritation testing beyond tissue viability assessment. Fucoxanthin proved to be non-irritant in RHS and already showed first skin protective effects following topical application.

## 1. Introduction

Epidemiological data and clinical presentation provide conclusive evidence for UV radiation as the major cause of skin aging, cancer, and inflammation [1,2]. UVB radiation promote the dimerization of pyrimidine DNA bases to cyclobutane dimers and subsequently C to T base transitions. Abundant C to T base transitions and CC to TT tandem mutations are referred to as a UVB signature or fingerprint. UVA radiation increases numbers of reactive oxygen species, which oxidize DNA bases to 8-hydroxyguanine, and cause G-to-T base transversion [3]. Thereby, the production of reactive oxygen species has two major causes. First, UV radiation directly oxidizes the major sebum component squalene and, thus, drives the expression of pro-inflammatory cytokines [4]. Second, UVA reacts with nitrogen oxides and volatile organic compounds, both abundant air pollutants in urban areas, to ozone [5]. Ozone itself does not penetrate the skin, but the ozone-mediated peroxidation of unsaturated lipids on the skin’s surface induces oxidative stress and inflammatory responses in deeper skin layers [6].

Prevention is considered as the prime strategy to reduce the number of skin cancer patients because treatment remains insufficiently effective in terms of cure rates and recurrence [7]. Yet, the widely recommended sunscreen application also provides insufficient protection against direct or indirect sunlight effects. Frequent underdosage [8] and abuse of sunscreens for intentional prolonged sun exposure [9] contribute to their low efficacy. Moreover, current sunscreens currently do not absorb VIS and IR light. This filter gap allows VIS and IR radiation penetrating deep skin layers and to produce 50% of the total amount of reactive oxygen species in skin [10]. Due to minor amounts of endogenous antioxidants in deeper skin layers, reactive oxygen species are hardly eliminated in the dermis [11] as well as in aged skin [12]. Taken together, even optimal application of currently existing sunscreen will not provide full protection from the effects of sunlight and air pollution, emphasizing the need for effective antioxidants in skin care.

Antioxidants address this issue by lowering the amount of reactive oxygen species. Administered either orally in nutraceuticals or topically in cosmetic products, antioxidants comprise carotenoids, squalene, and vitamins, to name but a few. Although nutraceuticals received considerable interest, studies on bioavailability, efficacy, and mechanism of action are scarce [13]. Furthermore, their uncontrolled use might cause secondary effects and interactions with medicinal products. Focusing on carotenoids, β-carotene and fucoxanthin are known for their antioxidant and cytoprotective functions and exemplarily represent the two carotenoid classes, carotenes and xanthophylls. Fucoxanthin was first isolated from the marine brown seaweeds and differs from other carotenoids due to an unusual allenic carbon (C-7′),5,6-monoepoxide, two hydroxyl groups, a carbonyl group, and an acetyl group in the terminal ring of fucoxanthin [14]. This particular chemical structure causes similar or higher antioxidant activities in comparison to those of α-tocopherol as well as suppressive effects on superoxide anion and nitric oxide generation [15,16]. Despite of the high antioxidant activity, oral fucoxanthin application does not result in efficient cutaneous concentrations. Fucoxanthin and its metabolites were primarily found in adipose tissue, liver, lungs, kidney, heart, and the spleen of mice [17]. Thus, a topical application of the lipophilic compound should achieve higher fucoxanthin doses at the target site.

The poor water solubility of fucoxanthin (log*P* 7.8) poses a challenge for the formulation of topically-used fucoxanthin products. Alkyl benzoate and ethanol are frequently used as solubilizers for highly lipophilic substances [18], and especially ethanol enhances the skin penetration [19].

Herein, we investigated the toxicological effects of fucoxanthin, dissolved either in alkyl benzoate or ethanol, on inflammation (interleukin-1α, 6, 8), homeostasis (epidermal growth factor receptor, small heat and shock protein beta 1), and metabolism (N-acetyltransferase 1) as well as on tissue viability in reconstructed human skin from primary human cells. To evaluate fucoxanthin effects, we selected pro-inflammatory cytokines IL-1α, IL-6, and IL-8, as well as EGFR, to study the beginning of re-epithelialization and HSPB1 to monitor the protective functions under stress conditions as well as NAT1, since fucoxanthin is totally deacetylated in the intestinal lumen.

## 2. Materials and Methods

### 2.1. Reconstructed Human Skin

The experimental procedures conformed to the principles of the Declaration of Helsinki and were approved by the ethics committees of Charité—Universitätsmedizin Berlin (EA1/081/13). Informed written consent was obtained from all the donors or their parent or legal guardian. The reconstructed human skin (RHS) was cultured in 24-well plates with primary human keratinocytes and fibroblasts (passage 3, pooled from three donors) from foreskin with ethical committee approval (EA1/081/13), and after parents had signed the written informed consent. We made the dermal compartment on day 01 by pouring 0.5 mL collagen I (Biochrom, Berlin, Germany) with 1.14 × 10^5^ normal human dermal fibroblasts into the insert (0.4 μm pore size; Millicell, Merck, Darmstadt, Germany). We seeded 3.7 × 10^5^ normal human keratinocytes onto the dermal compartment on day 2 and raised the constructs to the air-liquid interface on day 3. The culture medium was changed three times a week for seven days.

### 2.2. Test Substance Application

After the RHS were fully differentiated on day 10, we placed them into new 24-well plates containing 0.5 mL of fresh medium and performed the test according to OECD test guideline no. 439 [20]. In brief, 10 μL of the following test substances were applied for 15 min: phosphate-buffered saline (PBS, Sigma-Aldrich, München, Germany), 5% (*w*/*v*) sodium dodecyl sulfate (SDS, CAS-no: 151-21-3; Carl Roth, Berlin, Germany), 0.5% (*w*/*v*) all-*trans*-fucoxanthin (≥95% pure, CAS-no: 3351-86-8; Sigma-Aldrich), C12-15 alkyl benzoate (Crodamol™ AB, CAS-no 68411-27-8, Croda, Brazil), and ethanol (99.5%, CAS-no: 64-17-5, Merck, Germany). Subsequently, the constructs were washed 12 times with 0.5 mL PBS, dried with a sterile cotton swab, and placed into new 24-well plates with fresh medium for 42 h at 37 °C, 5% CO_2_.

### 2.3. Viability Assay

Constructs were incubated with the test substances for 15 min followed by a 42 h post-incubation period. RHS viability was determined by measuring the metabolic activity of the constructs after exposure and post-incubation using a colorimetric test according to OECD test guideline no. 439 [20]. The reduction of mitochondrial dehydrogenase activity was assessed via the decreased formazan production following incubation with MTT (3-(4,5-dimethylthiazol-2-yl)-2,5-diphenyltetrazolium bromide, Sigma-Aldrich). The formazan production was measured at 570 nm (FLUOstar OPTIMA, BMG Labtech, Ortenberg, Germany). We performed tests for interference of chemicals with MTT endpoint and correction in accordance to MatTek’s “In Vitro EpiDerm™ Skin Irritation Test” ([21], steps 1–4). The formazan readings were corrected by the fucoxanthin-related optical densities as well as by the optical densities due to direct MTT reduction and compared to those of negative control RHS [20]. Data are presented as the relative viability according to Equation (1):

(1) Relative viability (%)= 100 × [OD (test substance)/mean OD (negative control)]

### 2.4. Morphology and Immunofluorescence

Each RHS was snap frozen, sectioned into 8 µm slices (Leica CM 1510S, Wetzlar, Germany), and analyzed by hematoxylin-eosin or immunofluorescence staining. Antibodies against the following proteins were purchased from Abcam (Cambridge, UK): filaggrin (1:1000; ab81468), involucrin (1:500; ab111781), and from Dianova (Hamburg, Germany): keratin-10 (1:200, cat-no. AF0197-01). Pictures were taken with a fluorescence microscope (BZ-8000, Keyence, Neu-Isenburg, Germany) and analyzed by ImageJ software 1.52a [22].

### 2.5. Gene Expression

Real-time qPCR endpoint analysis was performed according to established procedures [23]. Briefly, the epidermal and dermal compartments of the RHS were mechanically separated. RNA from the epidermis was isolated using the NucleoSpin RNA II Kit (Macherey-Nagel, Düren, Germany). The TaqMan^®^ Reverse Transcription Kit (Thermo Fisher Scientific Inc., Waltham, MA, USA) synthesized cDNA by reverse transcription of 100 ng total RNA. Quantitative PCR was performed using the QuantStudio 5 Real-Time PCR System (Thermo Fisher Scientific) and the SensiFAST SYBR Lo-ROX Kit (Bioline, Luckenwalde, Germany) according to the manufacturer’s instructions. The primers were designed with UCSC Genome Browser [24] as described in Table 1 and were synthesized by EuroPrime (Invitrogen, Berlin, Germany). Gene expressions were normalized to the housekeeping gene ACTB.

### 2.6. Statistical Analysis

Data are presented as the mean + SD obtained from three independent experiments. Due to the explorative data analysis, a level of *p* ≤ 0.05 was considered to indicate a statistically significant difference. One-way ANOVA and subsequent Tukey post hoc tests were performed with GraphPad Prism 5.0 (La Jolla, CA, USA).

## 3. Results

### 3.1. Fucoxanthin Effects on RHS Morphology

**Figure 1:**
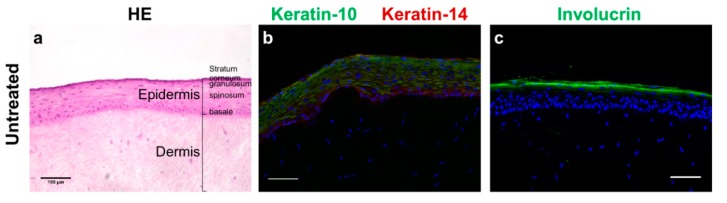
Morphology and protein expression in reconstructed human skin. (**a**) Hematoxylin-eosin staining showing all layers of human skin. (**b**) Immunolocalization of keratin-10 (green) in suprabasal epidermal layers and keratin-14 (red) in all epidermal layers. (**c**) Immunolocalization of involucrin (green), indicating terminal keratinocyte differentiation. Images are representative of three batches; nuclei in blue (DAPI); scale bars = 100 µm.

The 10-day culture of reconstructed human skin (RHS) resulted in a stratified epidermis with well-expressed *stratum basale*, *spinosum*, *granulosum*, and *corneum* (Figure 1a). Keratinocyte differentiation induced cell flattening and the expression of keratin-10 in suprabasal layers (Figure 1b). Keratin-10 and 14 was expressed throughout all epidermal layers (Figure 1b). Moreover, involucrin found in the *stratum corneum* showed the formation of a cornified envelope (Figure 1c). Although we observed also parakeratosis—an almost regular feature of skin models—and only small amounts of filaggrin, the RHS morphology suggested a sufficient skin barrier formation.

**Figure 2:**
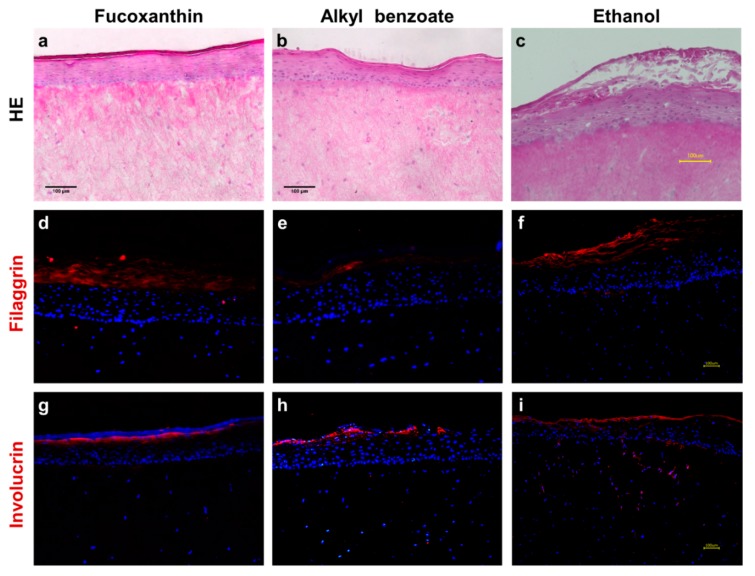
Morphology and protein expression in reconstructed human skin following fucoxanthin exposure versus the vehicles alone. (**a**–**c**) Hematoxylin-eosin staining with slightly disturbed morphology in c. (**d**–**f**) Immunolocalization of filaggrin with enhanced expression in d and f. (**g**–**i**) Similar immunolocalization of involucrin in all samples. Images are representative of at least three batches; nuclei in blue (DAPI); scale bars = 100 μm.

Neither fucoxanthin nor the vehicle alkyl benzoate disturbed tissue morphology (Figure 2a,b). Ethanol caused a detachment of the stratum corneum from the viable epidermis as well as slight damages in the coherence of the viable epidermis (Figure 2c). The filaggrin expression in RHS was slightly increased following exposure to fucoxanthin and ethanol (Figure 2d,f). Involucrin was homogenously distributed in the stratum corneum following all substance exposures (Figure 2g–i).

### 3.2. Fucoxanthin Effects on RHS Viability

Before assessing the RHS viability following substance exposure, we evaluated the interference of fucoxanthin with an MTT test. Fucoxanthin solutions as well as RHS treated with fucoxanthin were stained red, resulting in absorbance at 450 nm after isopropanol extraction although no MTT was added to these control tissues. Next, we investigated the direct reduction of MTT by fucoxanthin in solution as well as in freeze-killed tissues. Although we observed only a minor effect, we subtracted the absorbance due to fucoxanthin’s color and due to a direct interaction with MTT from all absorbance values of fucoxanthin-treated RHS in the viability tests. Phosphate-buffered saline (PBS) and sodium dodecyl sulfate (SDS) served as negative and positive controls, respectively, as recommended by the OECD.

**Figure 3:**
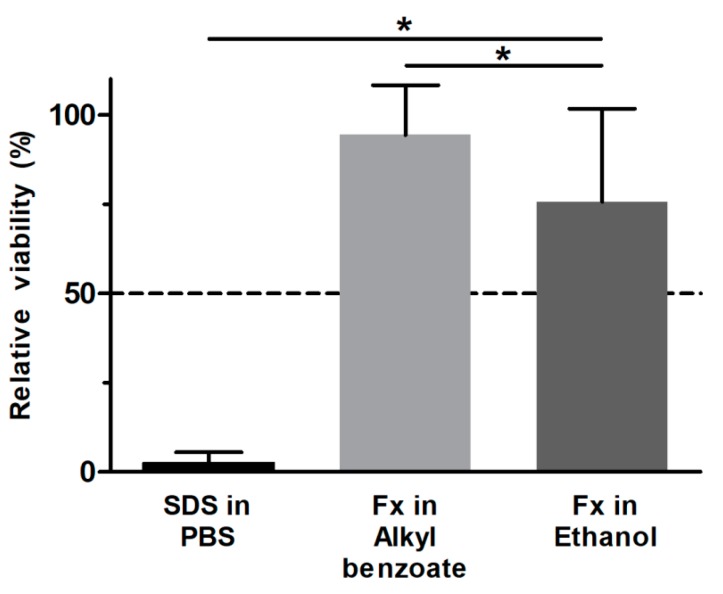
Fucoxanthin effects on the viability of reconstructed human skin. Test substances below the 50% threshold (dashed line) are predicted to be skin irritant. PBS—phosphate buffered saline (negative control); SDS—sodium dodecyl sulfate (positive control); Fx—fucoxanthin. Alkyl benzoate and ethanol were exposed for 15 min to the RHS and subsequently washed off. Mean + SD, *n* ≥ 3, * *p* ≤ 0.05 compared to SDS.

The viability of RHS following SDS exposure and 42 h post-treatment incubation period decreased to 2.6 ± 2.4%, correctly identifying SDS as skin irritant (Figure 3). Moreover, the standard deviation between tissue replicates fell far below 18%, and, thus, met the acceptability criteria of OECD test guideline no. 439.

Fucoxanthin showed a significantly higher viability than the positive control (Figure 3). Since the values exceeded also the threshold of 50%, fucoxanthin was not irritant to RHS. When testing the solvent controls, we observed a marked decrease in viability to 52.8 ± 9.0%. This tissue damage was ameliorated in the ethanolic test solution of fucoxanthin, as seen by a relative viability of 75.7%.

### 3.3. Fucoxanthin Effects on RHS Gene Expression

**Figure 4:**
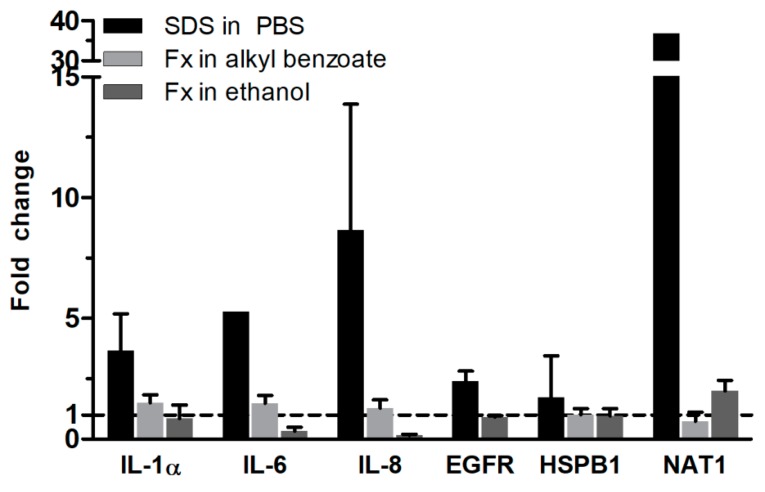
Fucoxanthin effects on the gene expression of reconstructed human skin. Fold change in gene expression was calculated in relation to the gene expression in reconstructed human skin exposed to the respective solvent (dashed line), SDS to PBS and Fx to alkyl benzoate or to ethanol. SDS—sodium dodecyl sulfate; PBS—phosphate-buffered saline; Fx—fucoxanthin; IL—interleukin; EGFR—epidermal growth factor receptor; HSPB1—small heat and shock protein beta 1; NAT1—N-acetyltransferase 1. Mean + SD, *n* = 3.

Next, we evaluated the gene expression following substance exposure and 42 h post-treatment incubation period to get a more detailed insight into the toxicity of fucoxanthin (Figure 4). SDS markedly increased the gene expression of N-acetyltransferase 1 (NAT1) as well as of pro-inflammatory genes, like interleukin (IL)-1α, 6, and 8, compared to the levels in PBS-treated RHS. Moreover, the gene expression of epidermal growth factor receptor (EGFR) and small heat and shock protein beta 1 (HSPB1) were slightly elevated.

Fucoxanthin exposure increased none of these gene expressions. When applied in alkyl benzoate solutions, we detected almost no change in gene expression. The ethanolic solution of fucoxanthin decreased the gene expression of IL-6 and 8 compared to the gene expression in ethanol-treated RHS to 33% or 15% compared to the solvent control samples. NAT1 gene expression was doubled compared to ethanol-treated RHS.

## 4. Discussion

Intrigued by the marked antioxidant efficacy of fucoxanthin [15,16], we investigated the potential toxicity of fucoxanthin in two different solvents, which are frequently used in dermatological products. Our results proved fucoxanthin to be non-irritant and suggested using alkyl benzoate as a solvent for the fucoxanthin (Figure 3). Absolute ethanol reduced the viability of RHS, but was necessary to dissolve the lipophilic fucoxanthin. Both viability testing and gene expression analysis revealed the ameliorating effects of fucoxanthin on the ethanol-induced inflammation in RHS (Figure 3 and Figure 4). Moreover, we proved the applicability of our novel 24-well-sized RHS, being fully compatible with organ-on-a-chip applications (Figure 1 and Figure 2). Using RHS extends the approach of OECD test guideline no. 439 and becomes mandatory when investigating biochemical pathways due to the known epidermal-dermal cross-talk in normal and diseased skin models [25,26].

The strong clinical need for skin protection is emphasized by the extraordinary increase of skin cancer patients. In Brazil, non-melanoma skin cancer accounts for 30% of diagnosed cancers [27], while in Australia, more people have been diagnosed with skin cancer than all other cancers combined [28]. The underlying biochemical mechanisms clearly provided the correlation between the cumulative exposure to UV radiation and skin cancer [3] and the contribution of VIS and IR radiation and air pollutants to cutaneous carcinogenesis has been described [6,10] as well. Aging accelerates a vicious circle of cumulative damage to the skin, reduced amounts of antioxidants among other age-associated conditions, like xerosis, impaired skin barrier, and wound healing, promoting the penetration of more pollutants into the skin [29]. The correlation of an additional 10 µg/m^3^ NO_2_ in the air with 25% more pigment spots on female cheeks [30] shows the cosmetic, and may indicate a medical, need for the prevention from extrinsic factors. However, protection from UV, or even from the entire spectrum of solar radiation cannot prevent from its detrimental effects, since nitric oxide, volatile organic compounds, and particulate matter also contributes to skin aging, inflammation, and cancer [5]. Thus, the strategy against skin aging must include a multitude of different approaches, including cleansing products to reduce the particle load on skin, agents that strengthen the skin barrier function, products that protect from sunlight, anti-inflammatory agents, and antioxidants [31].

Antioxidants from natural sources, like fucoxanthin, address these clinical and cosmetic needs due to the antioxidant properties. Herein, we exclusively investigated the effects of the commercially available fucoxanthin and not multi-compound algae extracts. Nevertheless, fucoxanthin needs to accumulate in sufficient amounts at the target site. The lipophilicity (log*P* 7.8) of fucoxanthin, as well as the accumulation of fucoxanthin and its degradation products in murine adipose tissue, liver, lungs, kidney, heart, and spleen [17], questions the efficacy of orally-administered fucoxanthin in skin. Thus, we applied the fucoxanthin solution topically to RHS and selected 0.5% as concentration, being in the range used for antioxidants in cosmetic formulations (0.01–1%). We dissolved the highly lipophilic fucoxanthin in alkyl benzoate, being devoid of genotoxic properties and frequently used in cosmetics as a solvent, emollient, preservative, and plasticizer [32]. Moreover, alkyl benzoate is already used to solubilize UV filters in sunscreens [18]. For comparison, we also included an ethanolic solution based on the recommendations for poorly water-soluble substances in skin irritation testing [33]. Moreover, the high ethanol concentration was required to dissolve fucoxanthin and allowed us to investigate the potentially ameliorating effects of this antioxidant.

Even the small amount of 10 µL ethanol solution per tissue, equal to 17 µL/cm^2^, disturbed the RHS morphology (Figure 2c), viability (Figure 3), and altered the gene expression (Figure 4). This is well in accordance with previous observations, where high concentrations were necessary for drug dissolution, as well [34]; more than 80% of cell death occurred even following the exposure to 50% ethanol in skin models [35]. The mechanism of tissue damage by ethanol is related to oxidative stress and well-known from the oral cavity [36]. Ethanol directly reacts to hydroxyethyl radicals and subsequently contributes to the formation of other ROS species. Chronic ethanol exposure causes CYP induction, mitochondrial damage, reduced antioxidant defense mechanisms, and thereby amplifies ROS-related tissue damage. Meanwhile, ethanol is also most effective in increasing the skin absorption of lipophilic drugs, like butenafine (log*P* 6.6) [37]. Assuming that ethanol also effectively increased the penetration of fucoxanthin (log*P* 7.8), fucoxanthin can counteract the pro-oxidative effects of ethanol. Thus, both ethanol and fucoxanthin toxicity data in our study are in accordance with previously-published results on polyphenols in grapes and red wine, which are suggested to protect from ethanol damage [38]. However, the efficacy of fucoxanthin will depend on the fucoxanthin concentrations within the tissue, as observed for other antioxidants.

The change in IL-6 and 8 gene expressions following test substance exposure correlates to the expression of IL-1α, HSPB1, and EGFR. IL-1α activates the p38 MAPK pathway to increase the expression of HSPB1, which causes anti-apoptotic effects, possesses chaperone-like activity and refolds denatured proteins, and is cytoprotective against heat shock [35]. EGFR also increases the expression of IL-8 [39] and plays an essential role in re-epithelialization by increasing keratinocyte proliferation and cell migration in wounded skin [40]. The constant gene expressions of EGFR and HSPB1 substantiate the absence of damage from fucoxanthin, but does not explain the reduction in IL-6 and IL-8 gene expression.

HSPB1 gene expression remained stable, even following the exposure to SDS. Although HSPB1 exerts protective functions under stress conditions [41], previous results with increased HSPB1 expressions were obtained 24 h after SDS treatment by Western blotting. Thus, the 42 h period of incubation was recommended for skin irritation testing [20], yet probably too long to detect maximum increase in HSPB1 gene expression.

Finally, we investigated the expression of NAT1 in RHS, since fucoxanthin is totally deacetylated in the intestinal lumen (for review see [14]). We observed an upregulation of NAT1 following the application of fucoxanthin in ethanol (Figure 4), indicating an activation of cutaneous metabolism, being relevant when applying drugs or cosmetic actives to the skin.

Instead of using murine models [42], we developed a full-thickness RHS to assess the potential toxic effects of fucoxanthin. Accumulating insights into the predictive power of animal-based tests in toxicology [43] emphasize the need for human cell-based models. Nevertheless, the skin model in this study consisted of primary juvenile cells and did not consider age-related changes in skin function. Future studies should investigate the efficacy of fucoxanthin in aged RHS [44], thereby taking age-related differences between young, middle-aged, and senior patients into account. Moreover, emerging markets for cosmetics demand the use of human cell-based test systems. For example, Brazil banned cosmetic products from being tested in animal models in 2019, but the Brazilian legislation gap in the use of biological material of human origin, which until recently impeded access to commercially-available skin models [45]. Thus, in-house or open-source protocols are urgently needed, but also need to be validated for their intended use. In the present study, we presented a fast (10-day culture) RHS protocol at reasonable price (3.7 × 10^5^ keratinocytes per construct), which is fully compatible to organ-on-a-chip applications. Multi-organ-chips provide the opportunity to study substance effects in an interconnected and perfused in vitro model, aiming to include the influence of liver function into substance evaluation [46].

## 5. Conclusions

Taken together, our study clearly demonstrated the non-irritancy of fucoxanthin. Fucoxanthin ameliorated detrimental effects of ethanol on tissue viability and inflammatory response. We observed metabolism activation by NAT1 upregulation, but no change in HSPB1 regulation. Finally, we proved the applicability of our novel, organ-on-a-chip compatible RHS for the evaluation of substance effects.

## Figures and Tables

**Table 1:** Primer sequences for PCR studies.

Gene Name	Used Primer Sequence	
	Forward	Reverse
**ACTB**	CCACCATGTACCCTGGCATT	GCTTGCTGATCCACATCTGCT
**EGFR**	GCCGACAGCTATGAGATGGAG	TGGAGGTGCAGTTTTTGAAGTG
**HSPB1**	GACCCCACCCAAGTTTCCTC	TCGGATTTTGCAGCTTCTGG
**IL-1α**	GTGACTGCCCAAGATGAAGACC	TGCCAAGCACACCCAGTAGTC
**IL-6**	CTGGATCAGGACTTTTGTACTCATCT	CCAATCTGGATTCAATGAGGAGACT
**IL-8**	GTGGAGAAGTTTTTGAAGAGGGC	TCTGGCAACCCTACAACAGAC
**NAT1**	ATCCGAGCTGTTCCCTTTGAG	AACATACCCTCCCAACATCGTG
